# An antioxidant ameliorates allergic airway inflammation by inhibiting HDAC 1 via HIF-1α/VEGF axis suppression in mice

**DOI:** 10.1038/s41598-023-36678-0

**Published:** 2023-06-14

**Authors:** Ramiya Islam, D. Dash, Rashmi Singh

**Affiliations:** 1 Department of Zoology, MMV, Banaras Hindu University, Varanasi, 221005 India; 2 Department of Biochemistry, Institute of Medical Sciences, Banaras Hindu University, Varanasi, 221005 India

**Keywords:** Drug discovery, Immunology, Diseases, Health occupations, Medical research, Pathogenesis

## Abstract

Histone deacetylase inhibitors (HDACi) are novel class of drugs as they are involved in post translational modification of several proteins involved in signaling pathways related to asthma. HDACi have been reported to elicit protective effects on asthma but the signaling pathways associated with it have not been investigated much. Recently, we have demonstrated that intranasal administrations of Pan-HDAC inhibitors, sodium butyrate and curcumin, which have effectively reduced asthma severity via HDAC1 inhibition in Ovalbumin induced mouse model. Present study aimed to investigate possible pathways by which curcumin and sodium butyrate may minimize asthma pathogenesis via HDAC 1 inhibition. Balb/c mice were exposed (sensitized and challenged) with Ovalbumin to establish allergic asthma model followed by pretreatment of curcumin (5 mg/kg) and sodium butyrate (50 mg/kg) through intranasal route. Effects of curcumin and sodium butyrate on HIF-1α/VEGF signaling through activation of PI3K/Akt axis has been investigated using protein expressions followed by chromatin immunoprecipitation of BCL2 and CCL2 against HDAC1. Molecular docking analysis was also performed to investigate effects of curcumin and butyrate on mucus hypersecretion, goblet cell hyperplasia and airway hyperresponsiveness. Augmented expressions of HDAC-1, HIF-1α, VEGF, p-Akt and p-PI3K were observed in asthmatic group which was suppressed in both the treatments. NRF-2 level was significantly restored by curcumin and butyrate treatments. Protein expressions of p-p38, IL-5 and mRNA expressions of GATA-3 were also reduced in curcumin and butyrate treatment groups. Our findings suggest that curcumin and sodium butyrate may attenuate airway inflammation via down regulation of p-Akt/p-PI3K/HIF-1α/VEGF axis.

## Introduction

Persistent inflammation in allergic disorders i.e. rhinitis, asthma etc. is maintained by multiple cytokines (IL-4, IL-5 and IL-13) released from Th2 cells followed by mast cell degranulation, infiltration of inflammatory cellsand excessive mucus secretion^1,2^. Intereukin-5 (IL-5) is essential for eosinophilic inflammation and its expression is regulated by GATA3 binding protein (GATA-3). The zinc finger transcription factor GATA-3, highly expressed in Th2 cells is activated upon phosphorylation by p38 mitogen-activated protein kinase (MAPK). GATA-3 is critical for differentiation of these cells^3,4.^^.^

It is believed that, during asthma, activated inflammatory cells like neutrophils, eosinophils, and macrophages release higher amount of ROS (Reactive oxygen species) which lead to enhanced oxidative stress, inflammation and tissue damage^5,6^. Once activated in response to oxidative stress, Nrf2 (Nuclear erythroid 2 p45 Related Factor 2) gets translocated to the nucleus, leading to transcriptional stimulation of target genes^7^.

Hypoxia signaling pathway is thought to be pro-inflammatory and pro-asthmatic, as elevated hypoxic response has been noticed in bronchial biopsies of COPD (chronic obstructive pulmonary disease) and asthmatic patients. It has been proposed that hypoxia inducible factor (HIF) plays an important role in human allergic airway diseases^8,9^.

In response to cellular oxygen levels, the transcriptional activator, HIF-1regulates gene expression^10^. HIF-1 consists of two subunits, HIF-1α and HIF-1β, although the β-subunit protein is expressed constitutively, the stability and transcriptional activity of the α-subunit is regulated by intracellular oxygen levels as well as oxygen independent regulation of HIF-1α expression involving various growth factors and cytokines^11,12^. In contrast to being regulated by oxygen-dependent mechanism, multiple studies have shown oxygen independent regulation of HIF-1α expression involving various growth factors and cytokines^12^.

Earlier studies have demonstrated immunomodulatory role of PI3K/Akt axis in progression of AHR, airway inflammation and vascular permeability via regulation of VEGF (vascular endothelial growth factor) expression mediated by HIF-1α^13^. PI3K (phosphoinositide 3-kinase), being involved in the recruitment, activation and apoptosis of different inflammatory cells, contributes in the asthma pathogenesis^14^. Involvement of PI3K has been shown in oxygen dependent or independent manner^15,16^. Akt, a kinase is an intermediate factor in PI3K pathway and VEGF is an endothelial cell specific peptide having crucial role in angiogenesis and neovascularization^17^. Additionally, VEGF is also reported as a prominent stimulator of Th2 inflammation and airway remodeling that’s why considered as a major inducer of asthma^18^.

HATs (histone acetyltransferases) and HDACs (histone deacetylases) are involved in the regulation of redox signaling and inflammatory responses^19,20^. It has been reported that different diseases, including cancer results due to abnormal acetylation and deacetylation^21–23^. Further, enhanced level of HDAC1, a member of class I HDAC family, has been reported in inflammation related diseases^24–26^. Till date,18 HDAC isoforms have been reported and are divided into 4 classes; class I (HDACs 1, 2, 3 and 8), class IIa (HDACs 4, 5, 7 and 9), class IIb (HDACs 6 and 10), class III (sirtuins; comprising SIRTs 1-7), and class IV (HDAC11)^27,28^. HDAC inhibitors (HDACi), are nascent class of medications which garner interest for their therapeutic effects in several diseases including arthritis, cancer, and asthma^29–31^. Various HDACi are at different stages of clinical trials, however, the side effects associated with synthetic HDACi are major impediment to their usage^32^. This prompted the exploration of naturally occurring HDACi with low adverse effects in addition to therapeutic effectiveness.

Curcumin, scientifically known as diferuloylmethane, is the active component of *Curcuma longa*, commonly known as turmeric^33^. It possesses many medicinal properties including anti-inflammatory^34^, antioxidant and anti-cancer properties^35^. Curcumin (CUR), a natural pan-HDACi having less toxicity, is safely used in the diet since ancient times. Sodium butyrate (SOB), one of the metabolic products of intestinal microbiota, can be considered as simplest Pan-HDACi^36,37^. The promising effects of HDAC inhibitors are widely investigated^38–40^, but the mechanism by which these HDAC inhibitors work, is still under investigation. Recently, we have reported, impact of curcumin and sodium butyrate in reducing airway remodeling and inflammation in allergic asthma by effectively inhibiting HDAC1^41^. Henceforth, in present study, one of the possible pathwaysare being explored by which these two pan-HDAC inhibitors might modulate airway inflammation by affecting HDAC1.

### Statistical analysis

**Figure 1:**
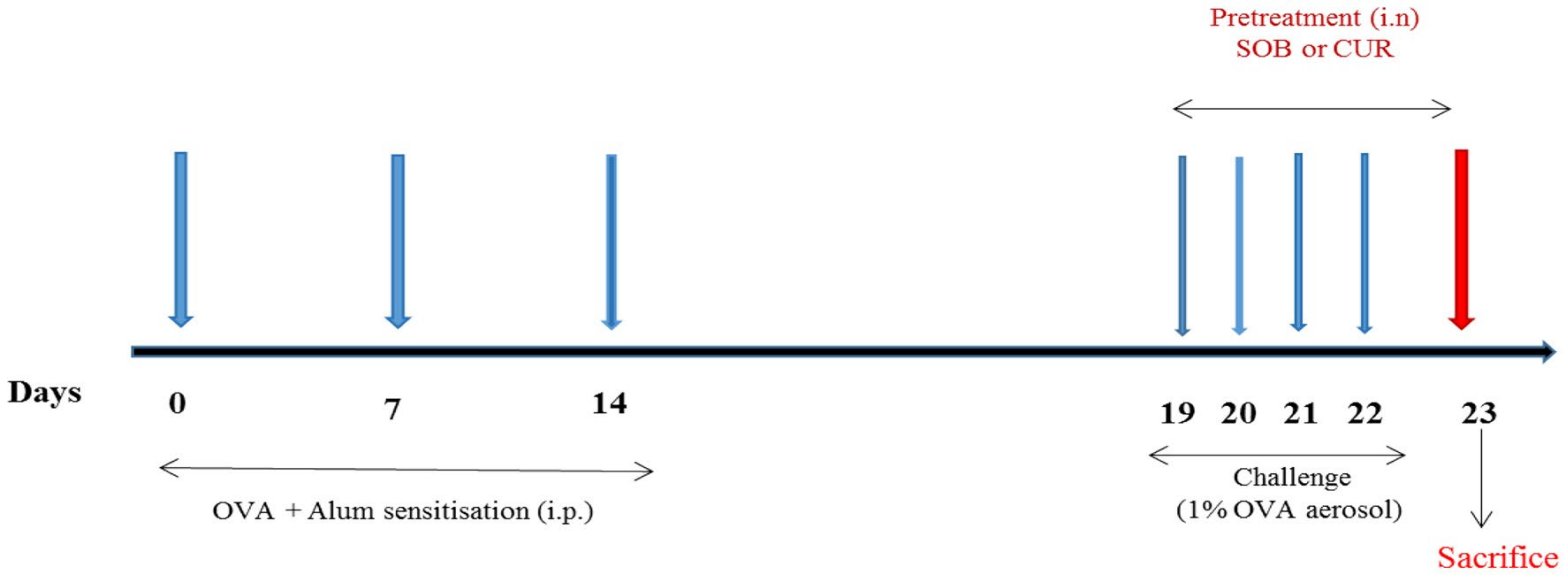
Sensitization, challenge and treatment schedule of animals. Mice were sensitized and challenged with OVA + Alum and OVA aerosol respectively and treated with SOB or CUR  as per experimental protocol.

The values are expressed as mean ± SE. Normal distribution was checked using QQ plots and Shapiro–Wilk tests. One-way ANOVA followed by Tukey's test was used to examine statistical significance and difference between the groupsusing SPSS 16. Statistical significance was considered at 5% level of significance (*p* 0.05). Three different iterations of the tests were conducted, and one representative set of results is shown here (Fig. 1).

## Results

### Effect of CUR and SOB on inflammation and ROS

**Figure 2:**
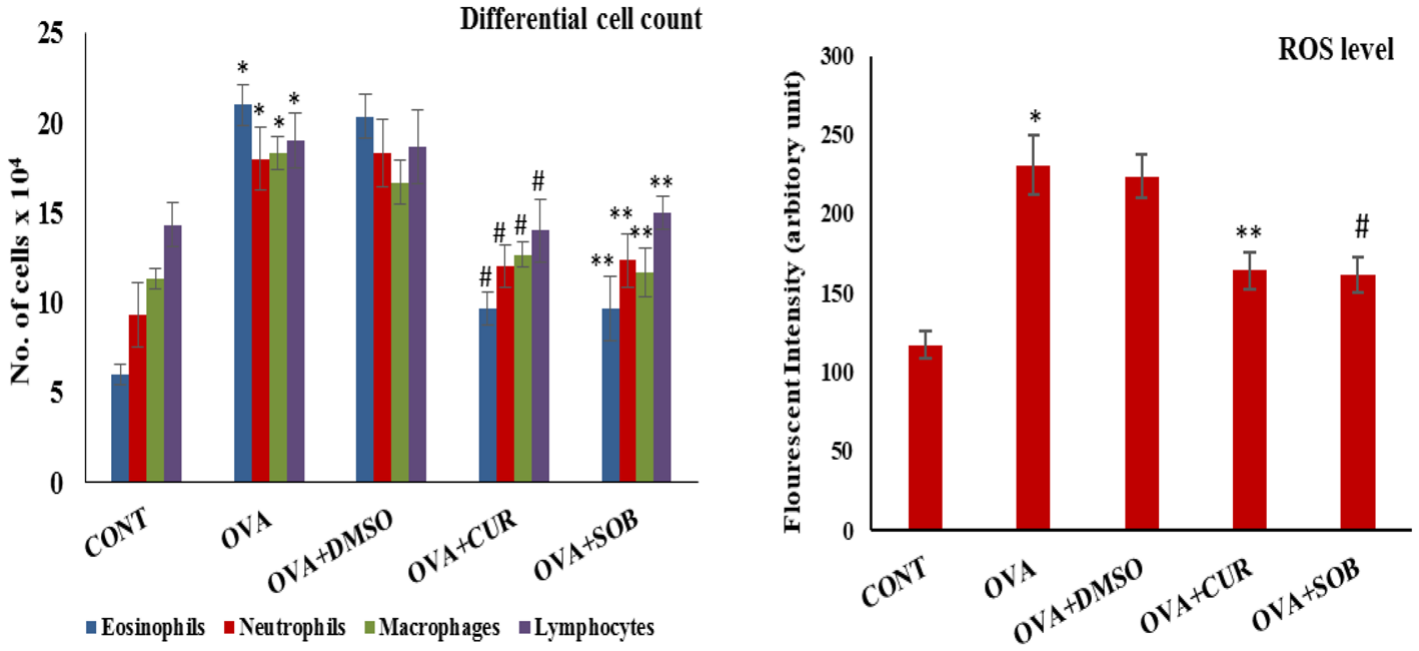
Differential cell count and ROS levels. Increased number of inflammatory cells and ROS levels were observed in OVA group as compared to control which was significantly decreased in CUR and SOB treated groups. Results are shown as means ± SE (*p* < 0.05) *CONT vs. OVA group, ** OVA vs. CUR group; # OVA vs. SOB group.

OVA Sensitization and challenge led to the significant infiltration of inflammatory cells to the lungs of asthmatic mice as compared to control. Inflammatory cells were identified on the basis of their nuclear morphologies and large number of inflammatory cells were noted in asthmatic group which was significantly reduced in treatment groups. Higher ROS level was found in OVA group as compared to control which was considerably reduced in CUR and SOB treatment groups (Fig. 2).

### Effects of CUR and SOB on oxidative stress

**Figure 3:**
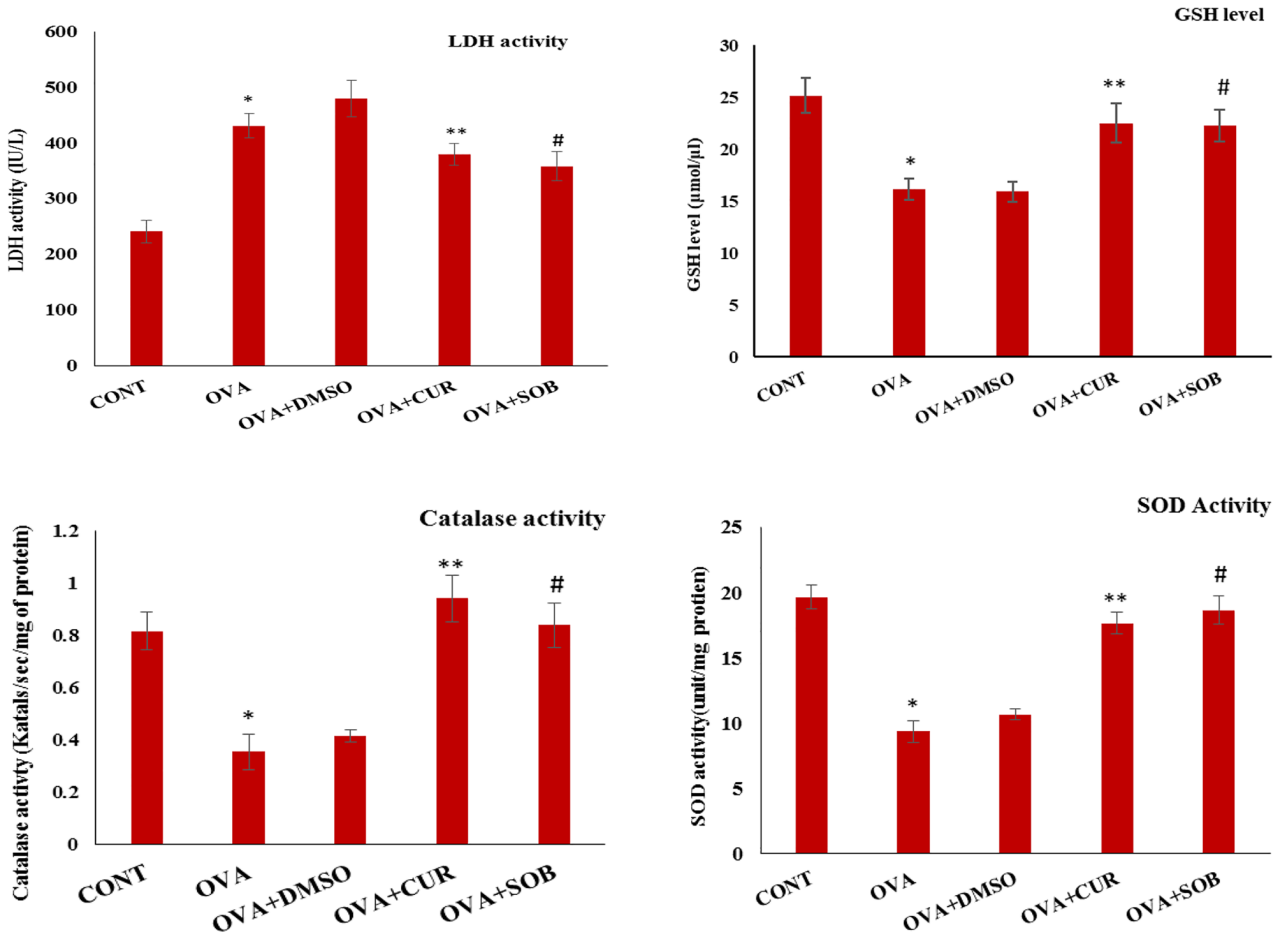
Assessment of LDH, GSH level and antioxidant enzyme activities in lungs. Increased LDH activity was observed in OVA induced group as compared to control which was significantly decreased by CUR and SOB treatment. Level of GSH and enzymatic activities of SOD and catalase was reduced in OVA induced group which was significantly restored in CUR and SOB treated groups. Results are shown as means ± SE (*p* < 0.05) ** OVA vs. CUR group; # OVA vs. SOB group.

Catalase and superoxide dismutase (SOD) are anti-oxidant enzymes present in cells while GSH (glutathione) is also involved in antioxidant response. OVA exposed oxidative stress was assessed by antioxidant enzyme levels. Reduced catalase and SOD enzyme levels were noted in OVA group as compared to control, whereas CUR and SOB has significantly restored enzyme levels (Fig. 3).

### Effect CUR and SOB on LDH levels

As compared to control group, higher LDH level was found in OVA induced asthmatic group which may be associated with lung damage whereas reduced LDH level was seen in both the treatment groups showing possible protective effect of CUR and SOB (Fig. 3).

### CUR and SOB restored NRF2 level

**Figure 4:**
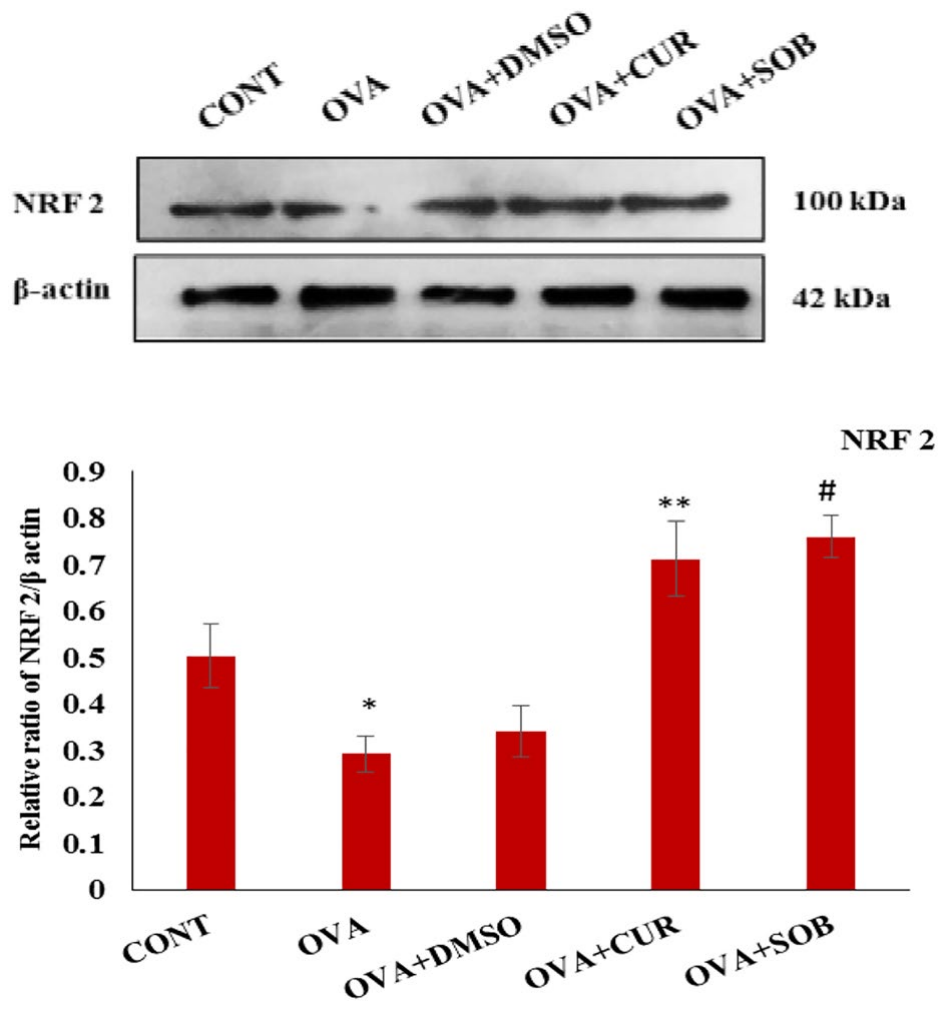
Protein expressions of NRF2 in lung tissue. NRF2 expression was significantly reduced in OVA group as compared to control and significantly restored in CUR and SOB treatment groups. Results are shown as means ± SE (*p* < 0.05) * CONT vs. OVA group, ** OVA vs. CUR group; # OVA vs. SOB group.

Transcription factor NRF2 is involved in antioxidant defense mechanism. Protein expressions of NRF2 were measured in lungs where reduced expression of NRF2 in asthmatic group was significantly restored in CUR and SOB treated groups (Fig. 4).

### Effect of CUR and SOB on p-p38, IL-5 and GATA-3 expressions

**Figure 5:**
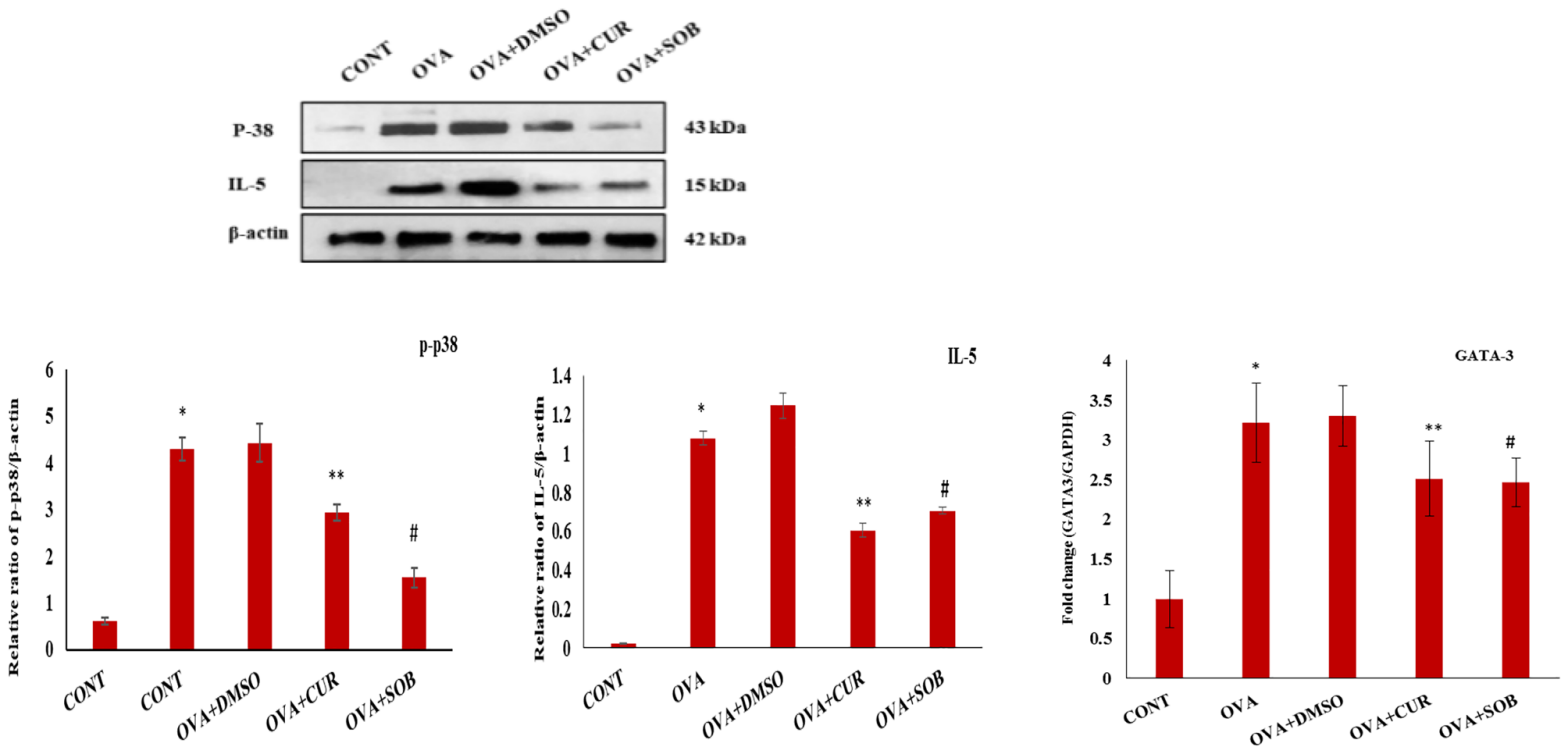
Protein expression of p-p38, IL-5 and mRNA level of GATA-3 in lung tissue. Expression of p-p38, IL-5 and GATA-3 were increased in OVA exposed asthmatic mice in contrast to control and was significantly reduced in CUR and SOB treated groups. GAPDH was used as reference gene. Results are shown as means ± SE (*p* < 0.05) * CONT vs. OVA group, ** OVA vs. CUR group; # OVA vs. SOB group.

GATA-3 is known to regulate IL-5 expression which is responsible for eosinophilic inflammation in asthma. Protein expressions of IL-5 and mRNA expressions of GATA-3 was checked by immune-blotting and RT-PCR respectively. Significant elevation in IL-5 and GATA-3 expressions were seen in OVA induced group as compared to control whereas significantly reduced expressions were observed in treated groups (Fig. 5).

### Effect of CUR and SOB on HDAC 1 expression

**Figure 6:**
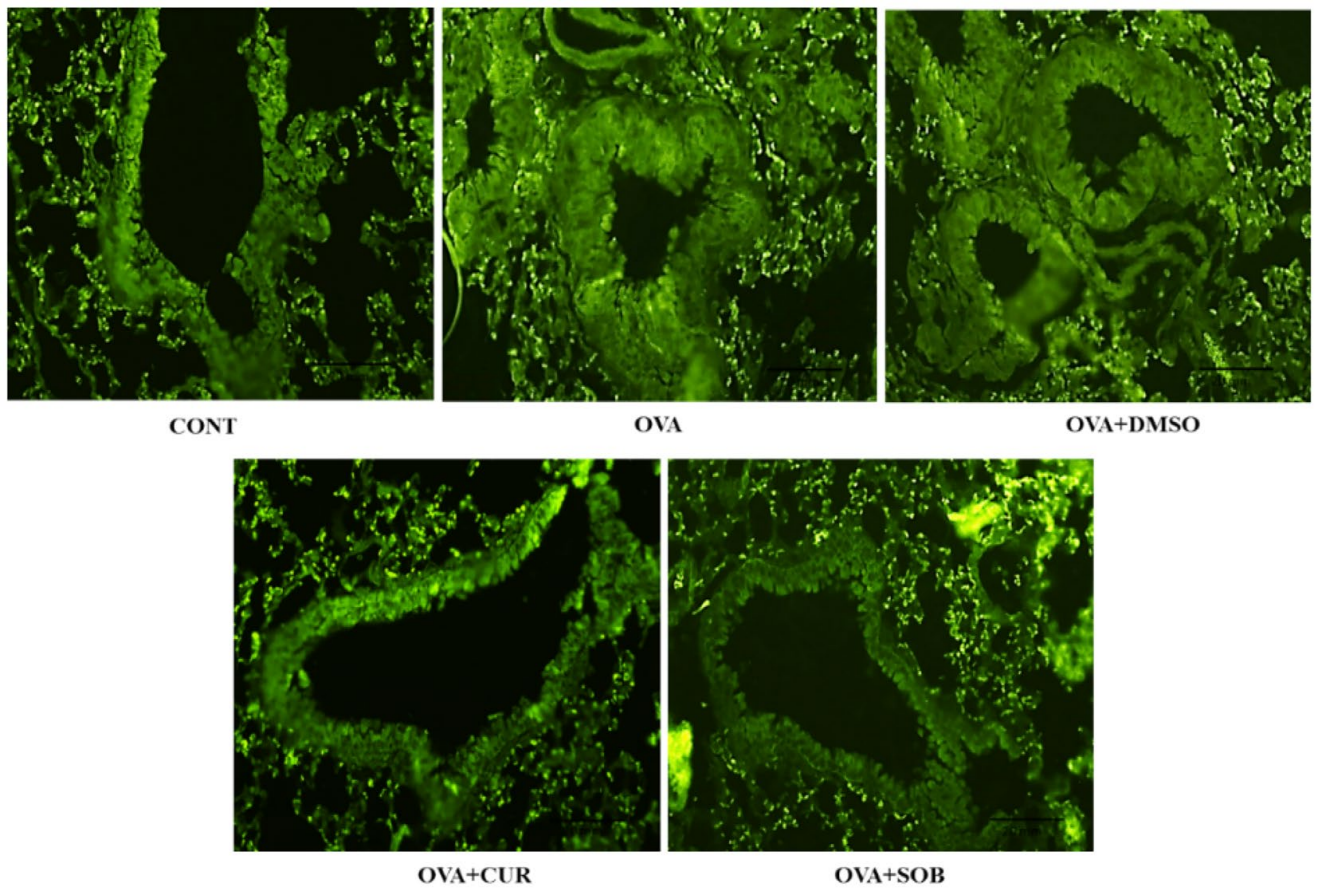
Expression of HDAC 1 in lungs detected by immunofluorescence. OVA exposure resulted in elevated expression of HDAC 1 in alveolar spaces and bronchioles (20 mm). However, marked suppression was observed in CUR and SOB treated groups.

**Figure 7:**
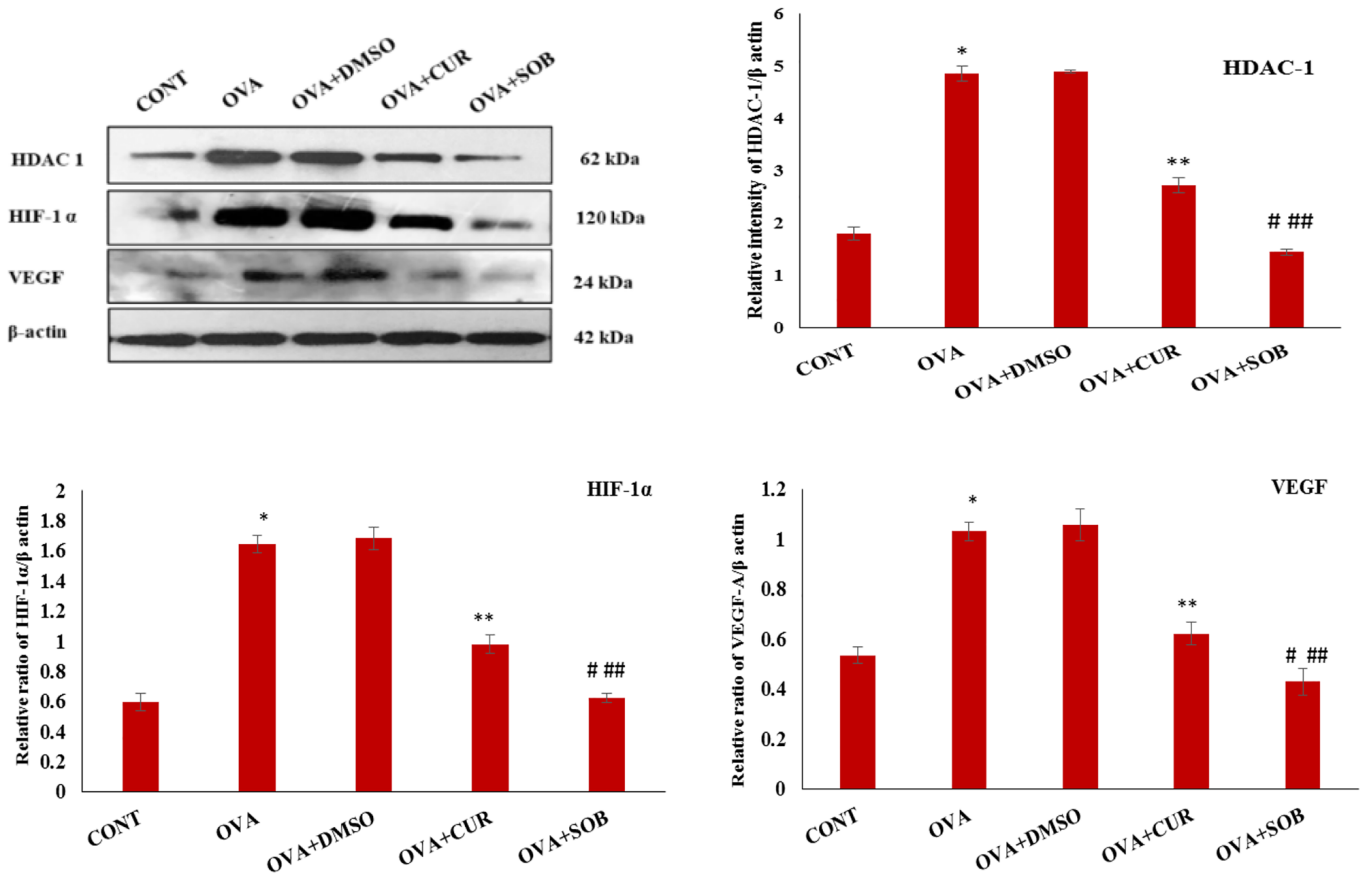
Protein expressions of HDAC-1, HIF-1 α and VEGF. Significant elevation was observed in HDAC-1, HIF-1 α and VEGF expressions in OVA induced mice whereas significant reduction was observed in CUR and SOB treated groups. Results are shown as means ± SE (*p* < 0.05) * CONT vs. OVA group, ** OVA vs. CUR group; # OVA vs. SOB group, ## CUR vs. SOB group.

Protein expressions of HDAC1 was measured in lungs by western blotting and immunofluorescence. Analysis of immunofluorescence and immune-blotting results revealed significant elevation in expressions of HDAC1 in lungs of OVA induced mice whereas significant reduction was observed in CUR and SOB treated group as compared to OVA and DMSO groups (Figs. 6, 7).

### CUR and SOB suppressed OVA induced VEGF and HIF-1α expression

To investigate effects of CUR and SOB treatment on vascular permeability and hypoxia conditions, protein expressions of HIF-1α and VEGF-α were analyzed (Fig. 7) which revealed increased expression of HIF-1 α and VEGF-α in asthmatic group whereas significant suppression was noted in CUR and SOB treatment groups.

### Effect of CUR and SOB on HDAC 1 binding at BCL2 and CCL2 promoters

**Figure 8:**
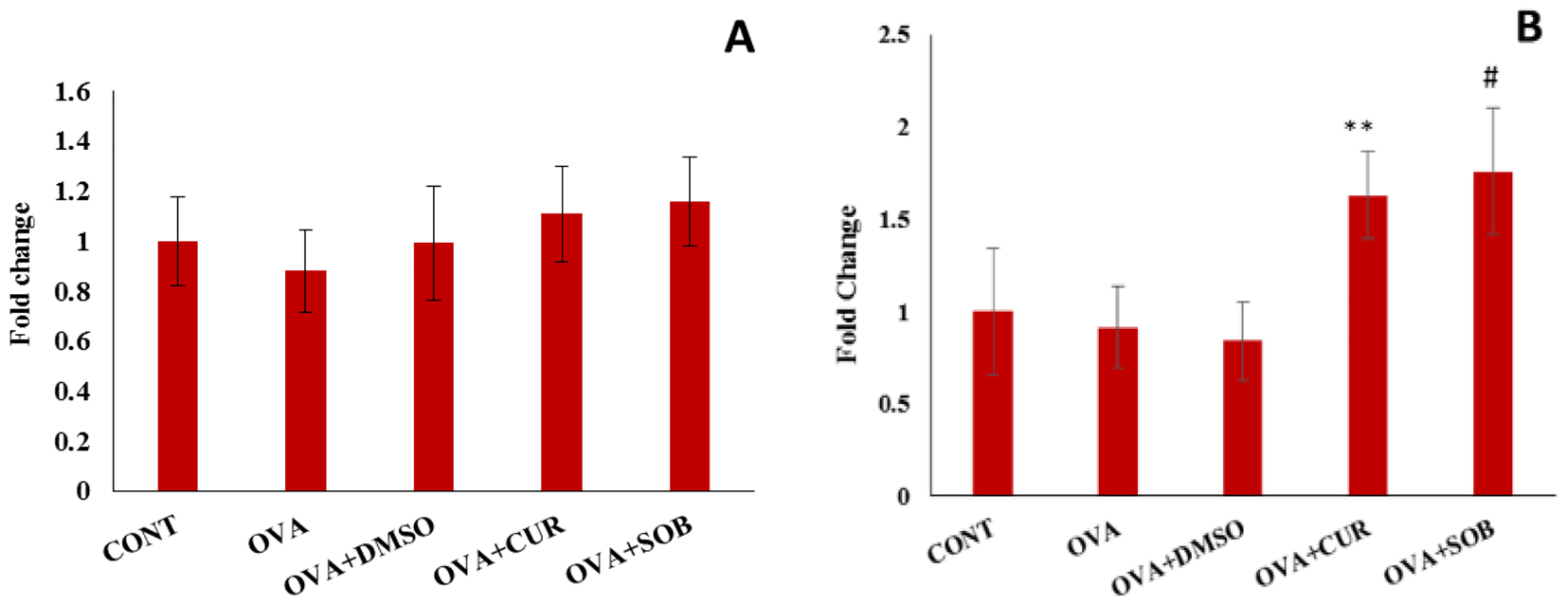
Binding of HDAC 1 at BCL2 and CCL2 promoters in lungs. Chip assay followed by qRT-PCR analysis showed no significant difference in HDAC1 and BCL2 interaction between the groups (**A**). Whereas at CCL2 promoter high HDAC1 binding observed in CUR and SOB groups (**B**). Results are shown as means ± SE (*p* < 0.05) * OVA vs. CUR group; # OVA vs. SOB group.

To find out possible mode of action of Pan-HDAC inhibitors by involvement of HDAC 1, we examined HDAC 1 specific BCL2 and CCL2 expression patterns using ChIP assay followed by qRT-PCR (Fig. 8). We found lower HDAC 1 binding at the promoter of BCL-2 in OVA induced mice as compared to control group which was enhanced in both the treatment groups. But the difference was not significant between the groups. However, at CCL2 promoter, high HDAC1 binding was observed in CUR and SOB treated groups.

### CUR and SOB suppressed activation of p-Akt and p-PI3Kaxis

**Figure 9:**
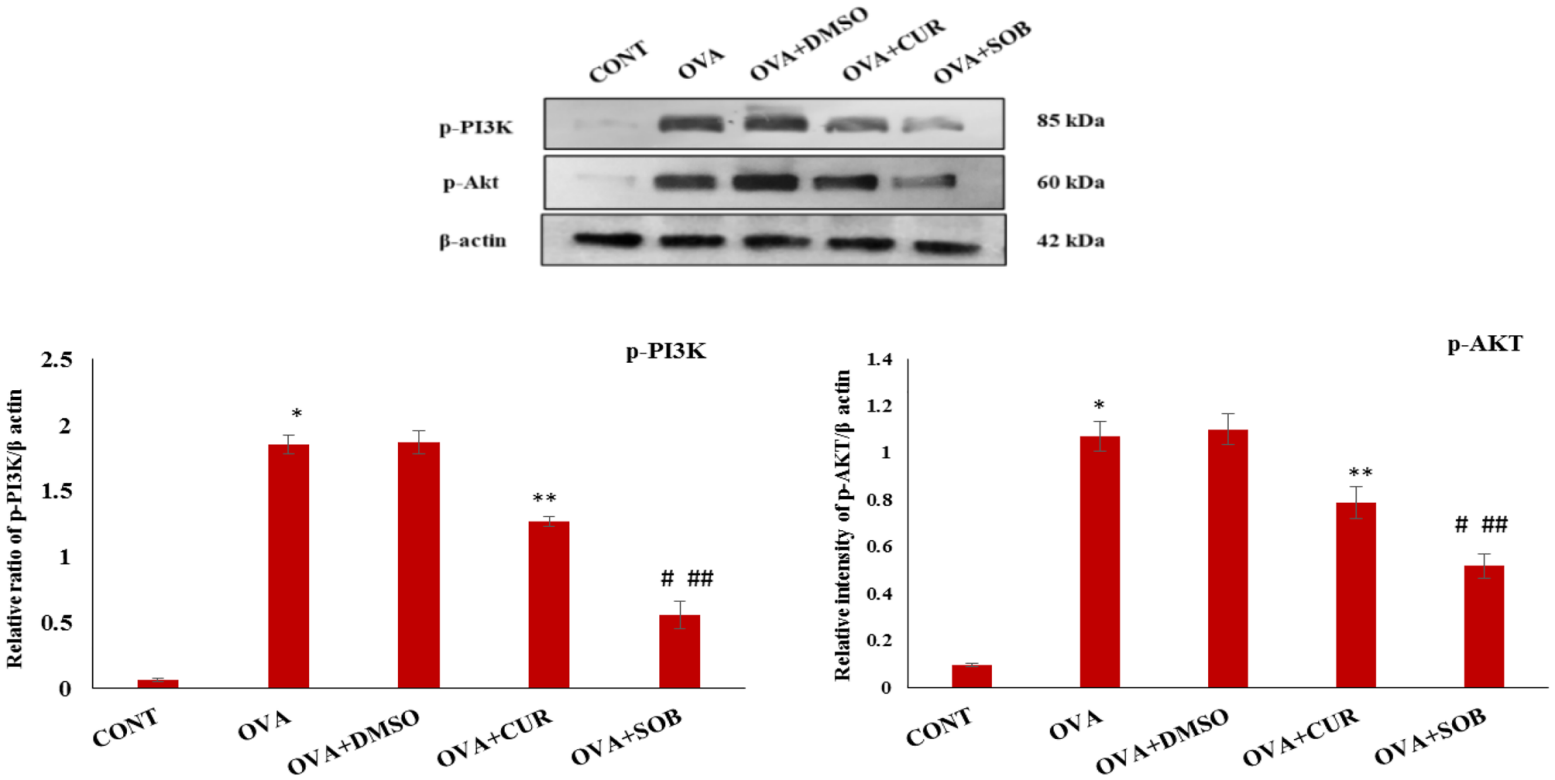
Protein expressions of p-AKT and p-PI3K in lung tissue. Significant elevation was observed in protein expression of these proteins in OVA induced mice whereas significant reduction was observed in CUR and SOB treated groups. Results are shown as means ± SE (*p* < 0.05) * CONT vs. OVA group, ** OVA vs. CUR group; # OVA vs. SOB group, ## CUR vs. SOB group.

To investigate inhibitory effects of CUR and SOB on activation of PI3K/Akt axis, protein expressions of p-Akt and p-PI3K were analysed in lungs (Fig. 9). Levels of phosphorylated PI3K (p-PI3K) and Akt (p-Akt) were significantly elevated in OVA induced asthmatic group as compared to control group which was significantly suppressed in CUR and SOB treated groups.

### Effects of CUR and SOB on AST and ALT levels

**Figure 10:**
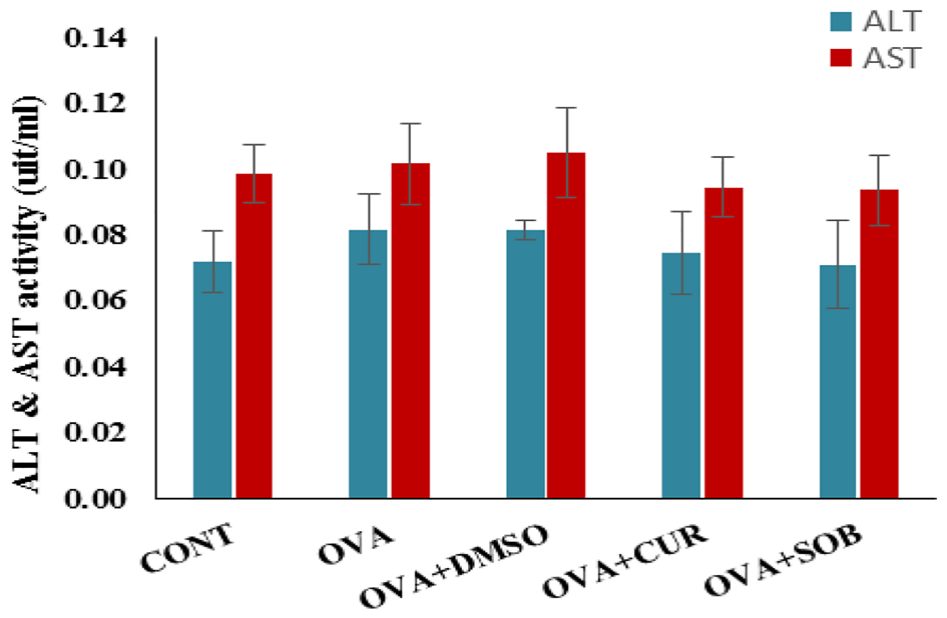
ALT and AST level measurement. No significant difference was observed between the groups in terms of ALT and AST levels.Results are shown as means ± SE (*p* < 0.05) ** OVA vs. CUR group; # OVA vs. SOB group.

To find out any possible cytotoxicity of bothCUR and SOB, ALT and AST levels, the indicators of hepatotoxicity were measured in serum. No significant change was observed in both the treatment groups (Fig. 10).

### In silico studies for effects of CUR and SOB on mucus hypersecretion, goblet cell hyperplasia and airway hyperresponsiveness

**Figure 11:**
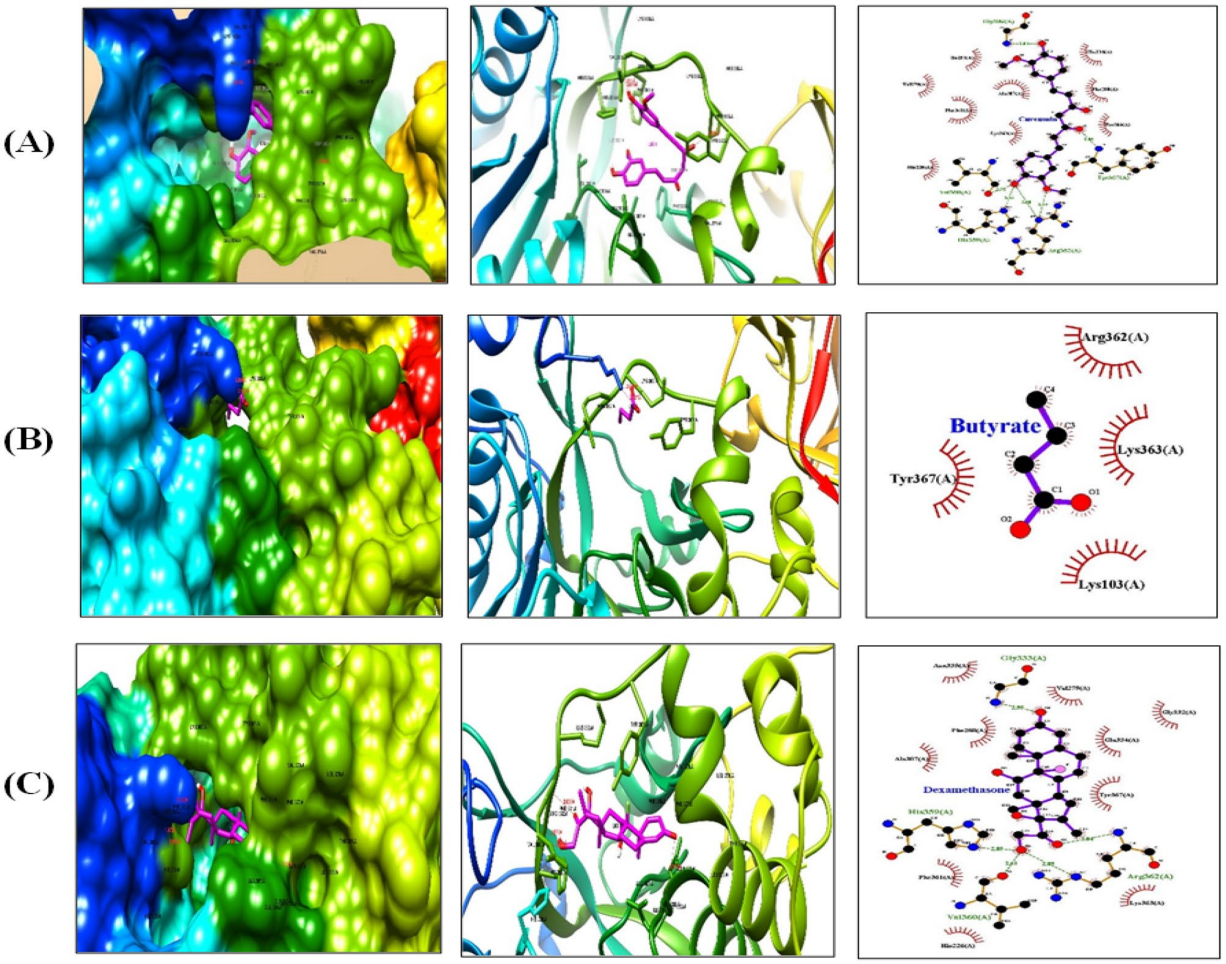
MUC5AC interaction diagrams with (**A**) CUR, (**B**) SOB and (**C**) DEXA shown by hydrophobic, ribbon and LigPlot imaging respectively.

**Figure 12:**
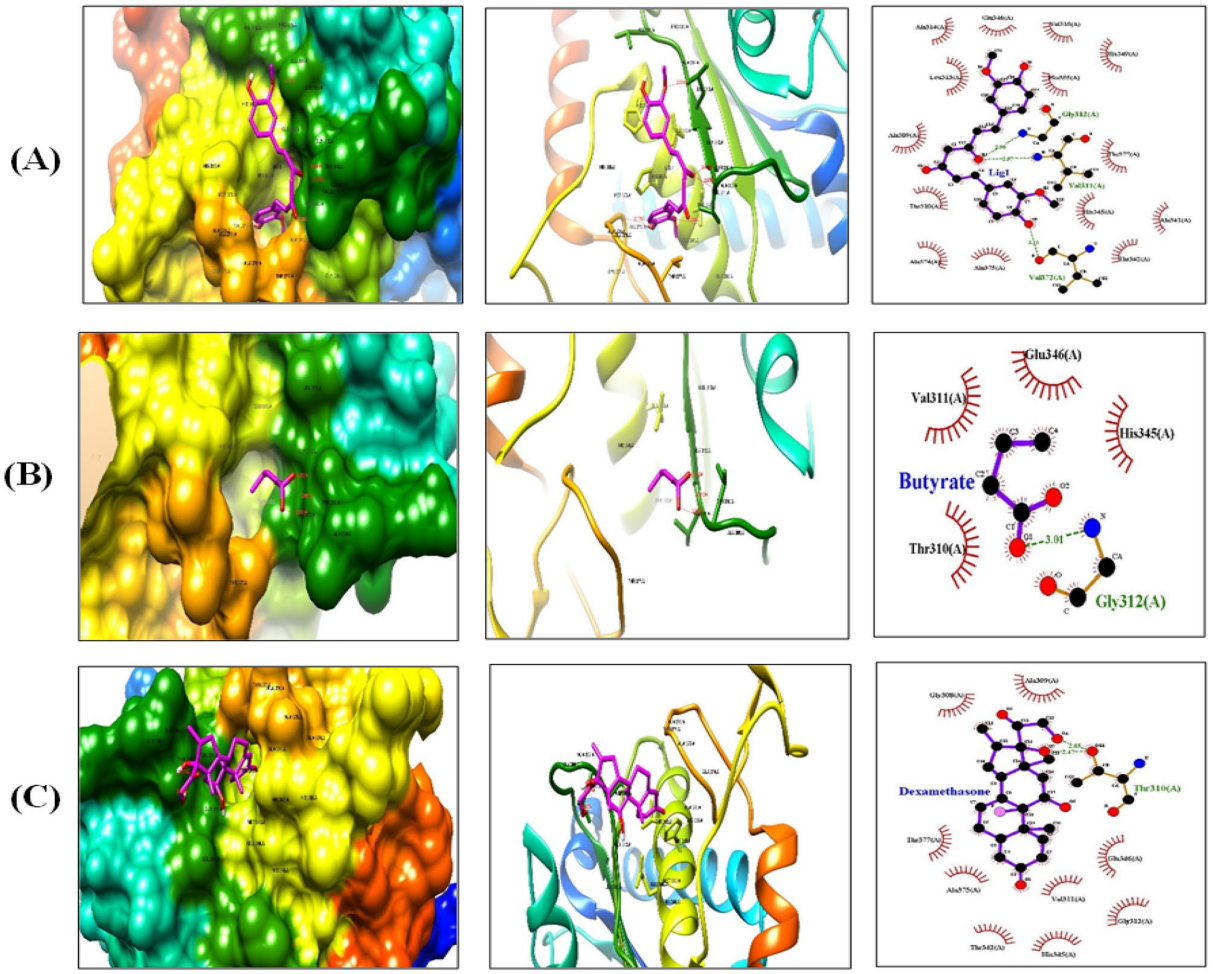
ADAM33 interaction diagrams with (**A**) CUR, (**B**) SOB and (**C**) DEXAby hydrophobic, ribbon and LigPlot imaging respectively.

The molecular docking studies of the ligands on the OvCHT1 model were carried out using AutoDock Tools (ADT). Protein–ligand interactions of CUR and SOB with MUC5AC, FOXA2 and ADAM33 were analyzed and compared with dexamethasone (DEXA) as reference drug. The binding energies and number of hydrogen bonds of docked compounds towards the target receptor are shown in Table 3. Among these, ligand curcumin showed best results for ADAM33 and with minimum binding energy − 7.46 kcal/mol. Here curcumin expressed better results than the reference drug. Butyrate also showed effective results in this docking study (Figs. 11, 12).

## Discussion

In present study, anti-inflammatory effects of CUR and SOB as HDACi was compared in OVA induced asthmatic model. Anti-asthmatic effects of CUR and SOB on inflammation and oxidative stress were determined by protein expression studies of HDAC 1, NRF2, p-p38, IL-5, and mRNA expression of GATA-3 were compared with histopathological analysis. To find out changes in HDAC 1 expression in OVA exposed and CUR/SOB treatment groups, ChiP assay was performed against HDAC 1 for BCL2 and CCL2 promoters. To investigate possible pathways involved, protein expressions of HIF-1α, VEGF, p-AKT and p-PI3K were checked along with LDH measurements and cellular oxidative stress studies. Protective effectsof CUR and SOB against asthma is consistent with our previous study where both have significantly minimized airway inflammation and remodeling^41^.

Allergic airway inflammation is characterized by infiltration of inflammatory cells, dominantly eosinophils^42^. Enhanced inflammatory cell infiltration (eosinophils, neutrophils, basophils etc.) was noted in OVA induced asthmatic mice in contrast to control as observed in total and differential cell counts in BALF cell pellet. Reduced inflammatory cell recruitments was seen in CUR and SOB treated groups (Fig. 2). In OVA induced asthmatic group, eosinophils are the major cells responsible for disease pathogenicity. These cells promote synthesis of collagen and fibrotic factors, hence play crucial role in fibrosis^43,44^.

Oxidative stress plays important role in initiation of hypoxia response and hypoxic environment is believed to promote oxidative stress thereby inflammation^45,46^. In asthmatic airways, the recruited and activated cells, i.e., eosinophils, monocytes, neutrophils and macrophages as well as epithelial cells have great potential for ROS production^47,48^. Enhanced ROS level in BALF was observed in asthmatic animals as compared to control which was significantly reduced by SOB and CUR treatments (Fig. 2).

SOD and catalase are antioxidant enzymes present in cells to manage the oxidative stress while reduced glutathione (GSH) is considered as important scavengers of ROS^49^. NRF2 is important for antioxidant defense mechanism which maintains cellular redox homeostasis. Diminished expression of NRF2 is related to severity in inflammation and asthma^50^. Low oxidative stress level is maintained by activation of NRF2, but higher oxidative stress level (ROS and RNS) led to initiation of pro-inflammatory signaling cascades^51^. We also observed lowered NRF2 expression in asthmatic group. Decreased activities of both the antioxidant enzymes and GSH, increased LDH levels in asthmatic group have shown the higher oxidative stress and cell damage in asthmatic condition (Fig. 3). NRF2 expression and activities of these anti-oxidant enzymes were significantly restored in CUR and SOB treatment groups (Fig. 4).

Involvement of HDAC 1in inflammatory and fibrotic conditions has been documented earlier. SOB as well as CUR are well known as natural pan-HDAC inhibitors and butyrate is known to inhibit class I and class II HDACs^52–54^.

Lately, researchers have suggested involvement of HDAC1 and HDAC3 in allergic inflammation-related diseases^24,25,55^. Further, involvement of HDAC 1 in inflammatory processes via multiple pathways like B cell proliferation, IFN-γ and HIF-1α mediated pathway has been reported antecedently^47,56,57^. We have also reportedearlier that OVA sensitization and challenge led to increased expression of HDAC 1 in OVA induced asthmatic mice lungs. Along with HDAC1, enhanced expressionof MMP-9, NF-kB and suppressed expression of H3acK9 was observed, which suggested probable relationship between these factors in allergic asthma pathogenesis^41^. Here western blotting and immunofluorescence staining results also confirmed higher expression of HDAC 1 in asthmatic group (Figs. 6, 7).

Studies have reported increased HIF-1α expression in asthmatic and rhinitis patients and suggested direct involvement of HIF-1α in allergic airway inflammation^58^. HIF-1α level can be upregulated via hypoxia independent mechanism or as a consequence of hypoxic microenvironment and in turn it also regulates expression of several factors such as pro-inflammatory cytokine, chemokines and adhesion molecules like VEGF. Furthermore, earlier reports suggested elevated expressions of HIF-1α, 2α and VEGF in BALF and bronchial epithelial cells of asthmatic patients which may be related to eosinophil^59,60^. Since higher eosinophil recruitments result due to higher level IL-5, therefore, we checked expressions of p-p38, IL-5 and GATA-3 in lungs. Our results are consistent with earlier findings where higher expression of p-p38, IL-5and GATA-3 were seen in OVA-induced asthmatic group (Fig. 5). Eosinophil recruitments were significantly reduced in both the treatment groups. Increased expressions of HIF-1α and VEGF was seen in asthmatic group as compared to control which appeared to near normal in CUR and SOB treatment groups (Fig. 7). Eosinophils are effecter cells in allergic inflammation and known to play important role in angiogenesis. VEGF is stored in granules of eosinophilsand released after IL-5 or granulocyte macrophage colony stimulating factor (GM-CSF) stimulations^18,61^. Large number of eosinophil infiltrations in hypoxic condition is accompanied by higher HIF-1α and VEGF expressions in asthmatic mice were noted which suggest important role of eosinophils in initiation of HIF -1α/VEGF activation. We propose here that OVA sensitization and challenge lead to activation of HIF -1α/VEGF axis via HDAC 1 upregulation where HDAC inhibitors CUR and SOB attenuates hypoxic response by inhibiting HDAC 1.

Monocyte chemotactic protein 1 (MCP-1) or CCL2 is a potent chemoattractant of macrophages and monocytes^62,63^ and due to their ability to recruit eosinophils, monocytes, activating mast cells and basophils, they may play significant role in asthma pathogenesis^64,65^. BCL-2 gene (B-cell lymphoma-2) is known to possess anti-apoptotic activity and are widely expressed in follicular non-Hodgkin’s lymphoma, hematopoietic malignancies and solid tumors^66^. Further, increased expression of BCL2 in eosinophils from sputum of asthmatic patients have been reported causing prolonged eosinophil survival whereas, expression of CCL-2 is found to be associated with HIF-1α expression in asthmatic airways^67,68^. Recruitment of HDAC 1 to the promoter of CCL2 has been reported in hepatic stellate cells. Similarly, it is also reported that DNA damage binding complex recruits HDAC 1 to repress BCL2 transcription in the human ovarian cancer cells indicating that higher recruitment of HDAC 1 is associated with repression of some genes^69^.

In line of these observations, we also hypothesized that HDAC1 is recruited to promoter site of CCL-2 and BCL-2. But we did not find higher binding of HDAC1 to BCL-2 promoter in either asthmatic or treatment groups (Fig. 8), instead, no significant differencewas observed among diseased and treated groups. Interestingly at CCL-2 promoter, higher binding of HDAC 1 was observed in SOB and CUR treated groups which was decreased in asthmatic group (Fig. 8). We found slightly lower binding of HDAC1 to BCL-2 in asthmatic group which might suggest higher expression of anti-apoptotic gene BCL-2 in asthmatic condition which supports earlier findings^67^. Similarly, higher binding of HDAC1 to CCL-2 was observed in SOB and CUR treated groups suggesting repression of CCL-2 by HDACi which also supports earlier reports^68^.

We assumed higher binding of HDAC 1 at both the promoters as protein expression of HDAC1 was increased in asthmatic group, but the results obtained is a paradox. HDAC1 binds differently at CCL-2 and BCL-2 promoters. The reason behind it could be due to the fact that HATs and HDACs have a variety of targets other than histones, moreover, they show pleotropic effects on various cellular processes^70^. Additionally, it has been suggested that HDACs act in a complex of multiple factors and expression and repression of a gene depends on the fact whether co-activator or co-repressor is recruited. Expression of HDAC1 in asthmatics is also a matter of contradiction. Taken together, we can state that, expression of genes mediated by HDACs, is dependent on the transcriptional activator or repressor complex in which, HDACs are a part^71–73^.

We next determined PI3K/Akt participation in HDAC1 mediated inflammatory response. PI3K being involved in asthma pathogenesis by dealing with recruitment, activation and survival of inflammatory cells. Furthermore, it has been suggested that PI3k activation is important for airway smooth muscle contraction and development of AHR in mice^74,75^. Enhanced expression levels of p-Akt and p-PI3K proteins was obtained in lungs of OVA induced mice which were significantly reduced in SOB and CUR treatment groups (Fig. 9) suggesting that inhibition of HDAC1 may regulate activation of PI3K/Akt axis. Earlier studies have also suggested that PI3K/Akt can modulate airway inflammation, AHR and vascular permeability through HIF-1α mediated regulation of VEGF in asthma pathogenesis^13,76^. Our findings suggest that HDAC1 may involve in hypoxia induced inflammation in allergic asthma and inhibition of HDAC1 by SOB and CUR reduces inflammation by inhibiting activation of PI3K/Akt axis.

Along with oxidative stress and inflammation, mucus hyper secretion, goblet cell hyperplasia and airway hyperresponsiveness are critical features of asthma that determine severity, morbidity and mortality in asthma. Mucin 5AC (MUC5AC), major constituent of mucus, is predominantly expressed by airway epithelial cells and its up regulated expression has been reported in chronic inflammatory diseases including asthma. Additionally, its expression is regulated by HIF-1α. Involvement of ADAM33 gene has been reported in airway hyperresponsiveness^77,78^. Molecular docking studies for MUC5AC and ADAM33 were performed and compared with DEXA to investigate possible effects of CUR and SOB on excessive mucous secretion and airway hyper responsiveness (Figs. 11, 12). Binding energies of CUR and SOB with above proteins were comparable to DEXA where CUR was found more effective (Table 3). Obtained results suggest that SOB and CUR may effectively reduce these pathological features of asthma.

## Conclusions

Based on the aforesaid data, it is proposed that HDAC inhibitors, SOB and CUR can ameliorate oxidative stress and airway inflammation in asthmatic mice by HDAC 1 inhibition via PI3K/Akt/HIF-1α/VEGF axis. CUR and SOB effectively restored the diminished level of NRF2 as well as antioxidant enzymes, thus mitigated the oxidative stress and hypoxic conditions in asthmatic airways. HDAC 1 may also be involved in regulating the expression of BCL 2 and CCL 2 during asthma. It is well known that most HDAC inhibitors exhibit pleiotropic cellular effects, making it challenging to pinpoint specific targets and determinetheir biological and clinical effects. Our findings suggest that pan-HDAC inhibitors, SOB and CUR possess effective therapeutic potential for asthmatics but further investigations are needed in order to understand the ramifications of HDAC inhibition.

## Material and methods

### Experimental groups

Balb/c mice (6–8 weeks old, 20 ± 2 g) were obtained from central animal facility of Central Drug Research Institute, Lucknow, India. Animals were housed and maintained under standard temperature condition at 25 ± 2 °C and 12 h light: dark cycle. Experimental animal handling and killing practices were approved by the Institutional Animal Ethical Committee Banaras Hindu University, Varanasi, India and all the experiments were performed in accordance with relevant ethical guidelines and regulations. Above study is reported in accordance with ARRIVE guidelines.

### Reagents

DCFDA (Dichlorflourodiacetate), Ovalbumin (OVA, grade V), Aluminum hydroxide (Alum) and Sodium butyrate were purchased from Sigma- Aldrich (St Louis, MO, USA). Antibodies against β actin, HDAC 1, NRF-2, p-Akt and HRP-conjugated secondary antibody were purchased from cell signaling technology, whereas RNAase A and ssDNA was purchased from Thermo Scientific (US). Antibody for p-PI3K was purchased from e-lab sciences, VEGF A was from Bioss USA. ALT and AST kits were purchased from recon and LDH (P-L) kit was obtained from Tulip, India. IL-5 and HIF-1α antibodies and Polyvinylidenedifluoride (PVDF) membrane were purchased from Genetix Biotech Asia Pvt. Ltd. whereas Immobilon western chemiluminescent HRP substrate kit was purchased from Merck (Darmstadt, Germany).

### Development of OVA induced Asthma model

Twenty five mice (Balb/c) were randomly divided into five groups (5mice/group) and named according to sensitization/challenge/ treatment protocol. Group I-control (CONT), Group II-asthmatic (OVA) (OVA + alum sensitized/OVA challenge/no treatment); Group III- OVA + DMSO (OVA + alum sensitized/OVA challenge/treated with DMSO i.n); Group IV-OVA + CUR, (OVA + alum sensitized/OVA challenge/ treated with CUR 5 mg/kg i.n.). Group V-OVA + SOB, (OVA + alum sensitized/OVA challenge/ treated with SOB 50 mg/kg i.n.) (Table 1). Control mice recieved 0.2 ml saline containing 4 mg aluminum hydroxide (alum) through intraperitoneal route and challenged with saline. On days 0, 7, and 14, the remaining four groups received injections of 0.2 ml saline solution containing 50 g of ovalbumin emulsified in 4 mg of aluminium hydroxide. Mice were exposed to 1% OVA aerosol (made in saline w/v) inhalation for 30 min daily from days 19 to 22 (Fig. 1)^77^.

**Table 1:** Grouping of animals. (*OVA* ovalbumin*, CUR* Curcumin) *SOB*)sodium butyrate, *DMSO* dimethysulphoxide, *i.n* intranasal, *i.p* intraperitoneal).

Sr. no.	Group	Sensitization (i.p)	Challenge (aerosol)	Treatment (i.n)
1.	Control	Saline	Saline	–
2.	OVA	OVA-alum	1% OVA	–
3.	OVA + DMSO	OVA-alum	1% OVA	DMSO
4.	OVA + CUR	OVA-alum	1% OVA	CUR (5 mg/kg)
5.	OVA + SOB	OVA-alum	1% OVA	SOB (50 mg/kg)

### Treatment schedule and experimental plan

To investigate the therapeutic potential of sodium butyrate and curcumin, each were administered separately as per doses 1 h before the OVA aerosol challenge. Mice were sacrificed by cervical dislocation following the last OVA aerosol challenge. Serum, BALF and lungs were collected and preserved for biochemical and histological examination.

### Collection of Bronchoalveolar Lavage Fluid (BALF), serum and lungs

After 24 h. of last OVA aerosol challenge, mice were sacrificed and BALF was obtained through trachea cannulation followed by washing off the airway lumen three times with 1 ml of ice-cold PBS. Lung washings were centrifuged at 3000 rpm for 10 min at 4 °C and cell pellet was used to study inflammation by differential cell count. BALF supernatant, serum and half lobe of lungs were preserved in − 80 °C whereas rest of the lung lobes were fixed in 10% neutral buffer formalin for histopathological examination.

### Differential cell count

Trypan blue dye exclusion test was used to determine total number of cells in BALF pellet. Cells were cytospun, fixed in methanol, and stained with geimsa for identification based on nucleus morphology. Different fields were chosen for identification and counting.

### Reactive oxygen species (ROS) measurement in BALF

BALF pellet was used for ROS measurement^78^. Briefly, in a 96 well black plate, 1 × 10^6^ cells were plated. After adding freshly prepared DCFDA (10 μM), the plate was incubated for 30 min at 37 °C in the dark. Fluorescence was measured in a micro plate fluorescence reader at excitation (485 nm) and emission (530 nm) wavelengths (Bio-Tek instruments Inc., 9 Winooski, VT, USA). ROS level was expressed as fluorescence intensity in arbitrary units.

### Catalase activity

Catalase activities in lung homogenate were determined using the previously described method^79^. In brief, lung homogenate was prepared and diluted in phosphate buffer (pH = 7.4) with H_2_O_2_ as a substrate. Catalase activity was measured in katal per second per mg of protein in each sample for 3 min at 240 nm.

### Super oxide dismutase (SOD) level

Superoxide dismutase (SOD) was assessed in lungs according to method described earlier^79^. In brief, 50 ul of lung homogenate was combined with 75 ul of 20 mM a-methionine, 75 ul of 100 mM hydroxylamine hydrochloride, 40 ulTriton x-100, and 100 ul 50 uM EDTA in phosphate buffer (pH 7.4). After 5 min of incubation at 37 °C, 50 uM riboflavin (80 ul) was added, and the reaction mixture was exposed to white light for 10 min. 1 ml Griess reagent (a 1:1 solution of 0.1% N-(1-naphthyl) ethylenediamine and 1.0% sulphanilic acid in 5% orthophosphoric acid) was added to the reaction mixture, and the absorbance at 543 nm was measured. SOD activity was calculated in milligrams of protein.

### Measurement of reduced glutathione (GSH) level in lungs

GSH level was determined using established protocol^80^. Briefly, lung homogenate (100 μl) was mixed with reaction mixture (600 μl) containing sodium phosphate buffer ((0.1 M, PH 7.0) and EDTA (1 mM). Further distilled water (760 μl) and DTNB (5,5-dithiobis (2-nitrobenzoic acid)) (40 μl, 0.4% w/v) prepared in 1% sodium tricitrate was added. After 5 min of incubation, absorbance was measured at 412 nm. GSH concentration was measured for each sample using a standard curve.

### Lactate dehydrogenase (LDH) level measurement in BALF

Lactate dehydrogenase is a cytoplasmic enzyme that is released into the extracellular fluid following cell injury. LDH levels in BALF were analyzed to assess lung injury using an LDH kit (Tulip) according to the manufacturer's instructions.

### Immunofluorescence

In brief, lung sections were deparaffinized, dehydrated and washed with PBS. After blocking with 10% goat serum for 2 h, sections were washed with PBST followed by incubation with HDAC 1 antibody (1:100) overnight. Further, sections were washed and incubated with fluorescein-tagged secondary antibody (1:400) for 2 h and mounted in Vectasheild mounting media (Vector Laboratories Inc, USA) containing 4,6 diamidino-2-phenylindole (DAPI) and analyzed under fluorescence microscope.

### Immunoblotting

In a homogenizing buffer containing a protease inhibitor cocktail, lung homogenate (10%) was prepared and centrifuged at 12,000 rpm for 20 min. Folin'sCiocalteu reagent was used to determine the protein content of supernatant. Proteins (30–50 g) were electrophoresed on SDS-PAGE (10–15%) and transferred on PVDF membrane in semidry transfer (Bio-Rad trans-Blot SD), followed by BSA or nonfat dry milk blocking. HDAC 1, NRF-2, p-p38, IL-5, p-AKT, p-PI3K, VEGF-A, HIF-1, and β-actin antibodies were used to probe the blot (as housekeeping gene). HRP-linked mouse anti-IgG secondary antibody and enhanced chemiluminescence kit were used to identify proteins. Image J software was used to evaluate gene expression after normalization with β-actin expression.

### Quantitative real time PCR for mRNA expression

Total RNA was extracted from mice lungs using Trizol reagent and converted (2 μg RNA) to cDNA using Qiagen reverse transcriptase kit according to manufacturer’s instruction. Specific primers for genes GATA 3^81^ and GAPDH^82^ were used to amplify using SYBR Premix Ex Taq master mix in ABI 7500 (Table 2). After normalizing the GAPDH mRNA level, the data were evaluated using the Ct (double delta Ct) method and displayed as fold change.

**Table 2:** List of primers.

Sr. no.	Gene	Sequence	Base pairs
1.	GATA-3 F	5′-AGGGACATCCTGCGCGAACTGT-3′	22
2.	GATA-3 R	5′-CATCTTCCGGTTTCGGGTCTGG-3′	22
3.	GAPDH F	5′CTCATGACCACAGTCCATGC′3	20
4.	GAPDH R	5′CACATTGGGGGTAGGAACAC′3	20
*ChIP specific primer sequence*			
5.	BCL2 F	5′-GTGGATGACTGAGTACCT-3′	18
6.	BCL2 R	5′-CCAGGAGAA ATCAAACAGAG-3′	20
7.	CCL2 F	5'-ATGTGAGAGCGCCACTCTTT-3'	20
8.	CCL2 R	5'-TGGTAGCTCTCTGCCCTGTT-3	20

### Chromatin immunoprecipitation

Chromatin immune-precipitation was performed as mentioned earlier^83^. Briefly, the lung tissue was minced in PBS and cross-linked with 1% formaldehyde at room temperature for 15 min. It was homogenized in protease inhibitor containing RIPA buffer, incubated at 4 °C for 5 min, and then centrifuged at 1000 g for 5 min. After being resuspended in nucleic acid lysis buffer, which contains 10 mM EDTA (pH 8.0), 1% SDS, 50 mMTris-Cl (pH 8.0), and 1 mM protease inhibitors, the pellet was incubated at 4 °C for 20 min. It was subjected to five cycles of sonication at 40 °C with a pulse duration of 30 s at 50% output. Bradford method was used to quantify the sonicated chromatin^84^. 250 µg of chromatin was incubated with Protein A-agarose bead slurry and single stranded Salmon sperm DNA for 4 h at 4 °C and then centrifuged at 14,000 × g at 4 °C for 10 min. The supernatant was divided into input and immunoprecipitation fraction. HDAC 1 antibody was added to immunoprecipitation fraction and incubated overnight. Next day, Protein A-agarose bead slurry was added to it and allowed to form bead/antibody/chromatin complex for 4 h. After centrifuged at 3000 × g for 10 min at 4 °C, the pellet was washed in low salt, high salt, LiCl, TE buffer and the immune complex was eluted by elution buffer. For reverse cross-linking of protein- DNA complex, 200 mMNaCl and RNase A was added to the pellet, incubated overnight at 65 °C in water bath and then DNA was isolated by phenol chloroform method. Using this eluted DNA as template, promoters were amplified with specific primers for CCL2^85^ and BCL2^86^ (Table 2). The fold change in gene expression was normalized with input control and was calculated by DDCt method.

### Measurement of alanine aminotransferase (ALT) and aspartate aminotransferase (AST) levels in serum

To investigate the toxicity of intranasal curcumin and sodium butyrate, the liver function test in serum was checked. Standard kits (Avecon) were used to measure the levels of the enzymes aspartate aminotransferase (AST) and alanine aminotransferase (ALT) by a modified Reitman and Frankel's colorimetric DNPH method (Table 3).

**Table 3:** Binding Energies obtained by molecular docking analysis.

Ligands	ADAM33	H-Bonds	MUC5AC	H-Bonds	FOXA2	H-Bonds
CUR	− 7.46	5	− 9.09	3	− 5.77	1
SOB	− 2.52	3	− 4.18	2	− 4.46	2
DEXA	− 7.30	2	− 9.64	4	− 4.49	3

## Supplementary Information

Supplementary Information.

## Data Availability

The datasets generated during the current study are available from the corresponding author on reasonable request.
