# Author Correction: An antioxidant ameliorates allergic airway inflammation by inhibiting HDAC 1 via HIF-1α/VEGF axis suppression in mice

**DOI:** 10.1038/s41598-023-37787-6

**Published:** 2023-06-29

**Authors:** Ramiya Islam, D. Dash, Rashmi Singh

**Affiliations:** 1 Department of Zoology, MMV, Banaras Hindu University, Varanasi, 221005 India; 2 Department of Biochemistry, Institute of Medical Sciences, Banaras Hindu University, Varanasi, 221005 India

Correction to: *Scientific Reports* 10.1038/s41598-023-36678-0, published online 14 June 2023

In the original version of this Article Rashmi Singh was incorrectly affiliated with ‘Department of Biochemistry, Institute of Medical Sciences, Banaras Hindu University, Varanasi 221005, India’. The correct affiliation is listed below.

Department of Zoology, MMV, Banaras Hindu University, Varanasi 221005, India.

The original Article has been corrected.

