# Supplementary material for: An antioxidant ameliorates allergic airway inflammation by inhibiting HDAC 1 via HIF-1α/VEGF axis suppression in mice
Source: Sci Rep. 2023 Jun 14;13:9637. doi: 10.1038/s41598-023-36678-0 (PMC10267105; doi:10.1038/s41598-023-36678-0)
Supplement: Supplementary file 1 — Supplementary Information. [file 41598_2023_36678_MOESM1_ESM.docx]

**An Antioxidant ameliorates allergic airway inflammation by inhibiting HDAC 1 via HIF-1α/VEGF axis suppression in mice**

**Ramiya Islam^1^, D Dash^2^ and Rashmi Singh^1^***

**SUPPLEMENTARY FIGURES**

**Supplementary Figure S1**

**Supplementary Figure S1**: Original whole western blot of NRF-2 and β-actin. The 6^th^ lane in the β-actin blot (from left to right, SoB+Cur) was an additional group which was discontinued due to technical reasons and not discussed in the manuscript. For the final figure, the bands were cropped and adjusted so that the bands appear better. The whole horizontal line of bands was adjusted. They were then placed in the Figure 4.


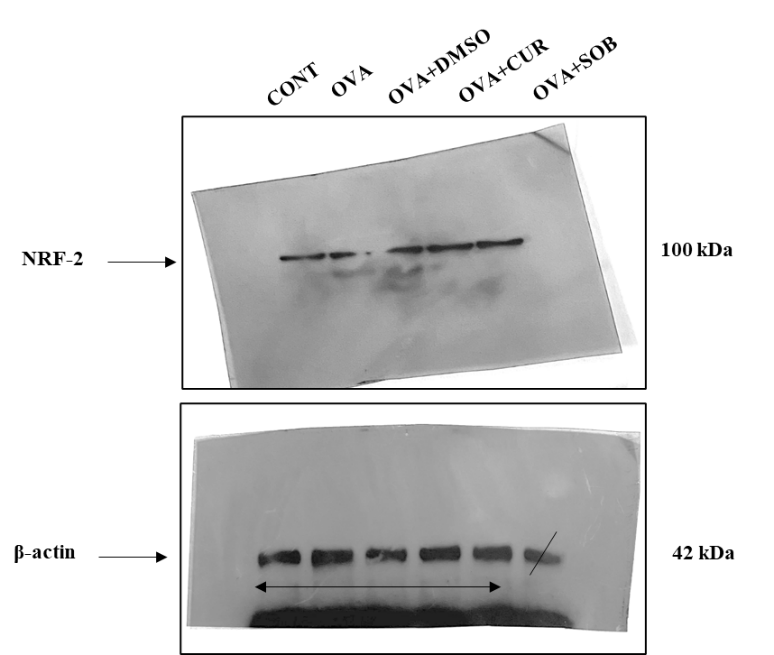


**Supplementary Figure S2**

**Supplementary Figure S2:** Original whole western blot of p38, IL-5 and β-actin. The 6^th^ lane in the IL-5 and β-actin blot (from left to right, SoB+Cur) was an additional group which was discontinued due to technical reasons and not discussed in the manuscript. For the final figure, the bands were cropped and adjusted so that the bands appear better. The whole horizontal line of bands was adjusted. They were then placed in the Figure 5.


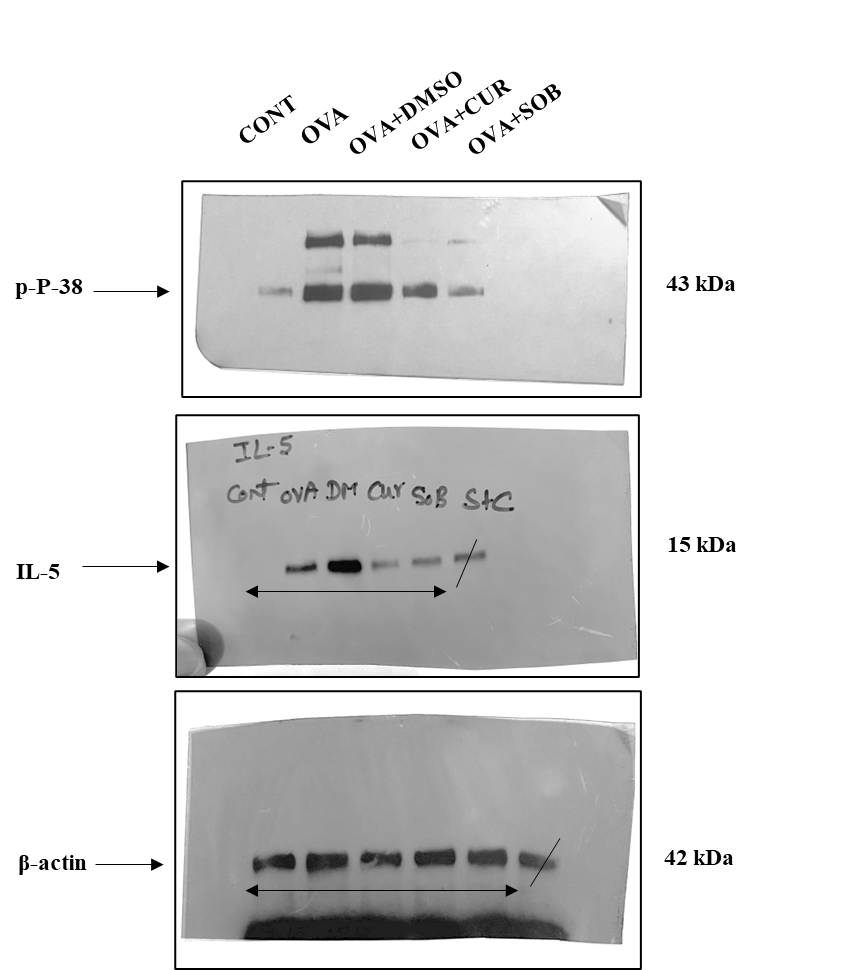


**Supplementary Figure S3**

**Supplementary Figure S3**: Original whole western blot of HDAC-1, HIF-1α, VEGF and β-actin. The extrabands in the blots were due to nonspecific binding (with protein ladder in β- actin blot) and (from left to right, Sob+Cur in HIF and VEGF blots) was an additional group which was discontinued due to technical reasons and not discussed in the manuscript. Membranes of HDAC 1 and VEGF were cut prior to incubation with primary antibodies and lower part was used for incubation with other antibodies for other proteins of interest. For the final figure, the bands were cropped and adjusted so that the bands appear better. The whole horizontal line of bands was adjusted. They were then placed in the Figure 7.


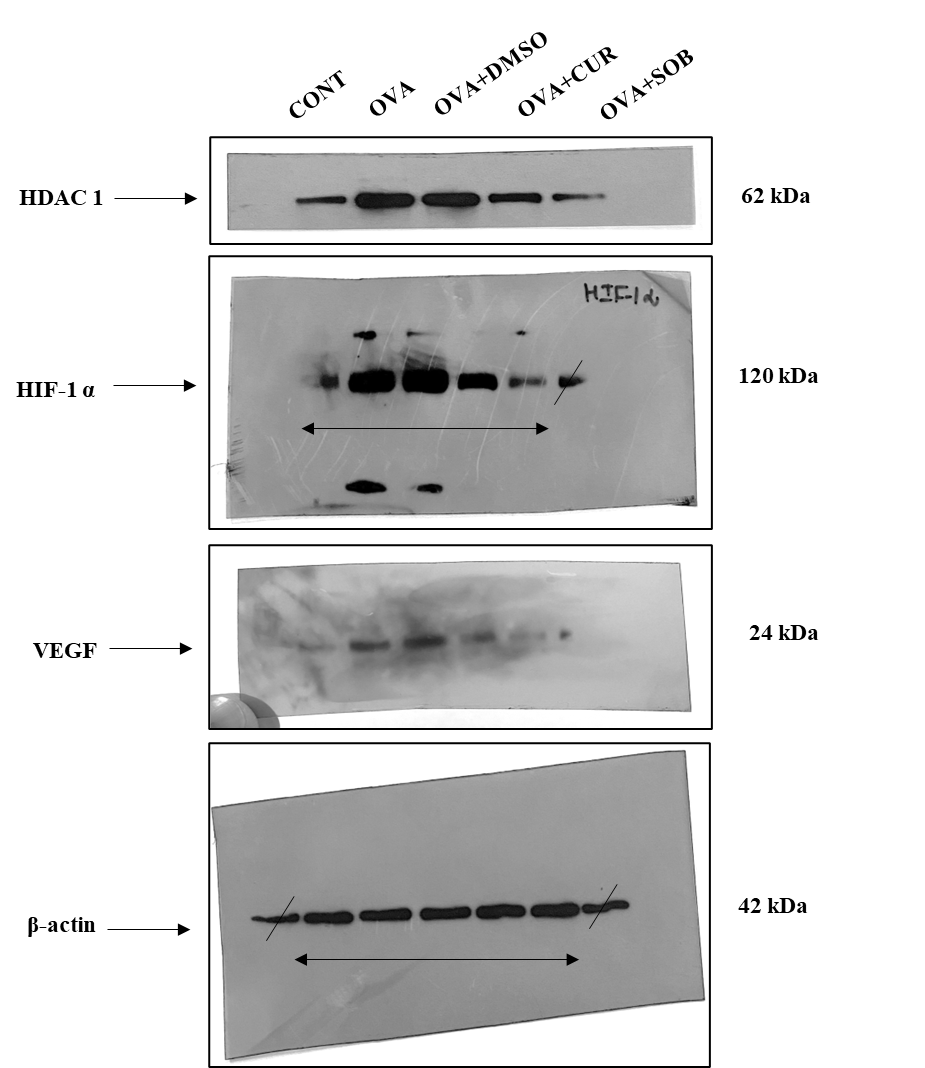


**Supplementary Figure S4**


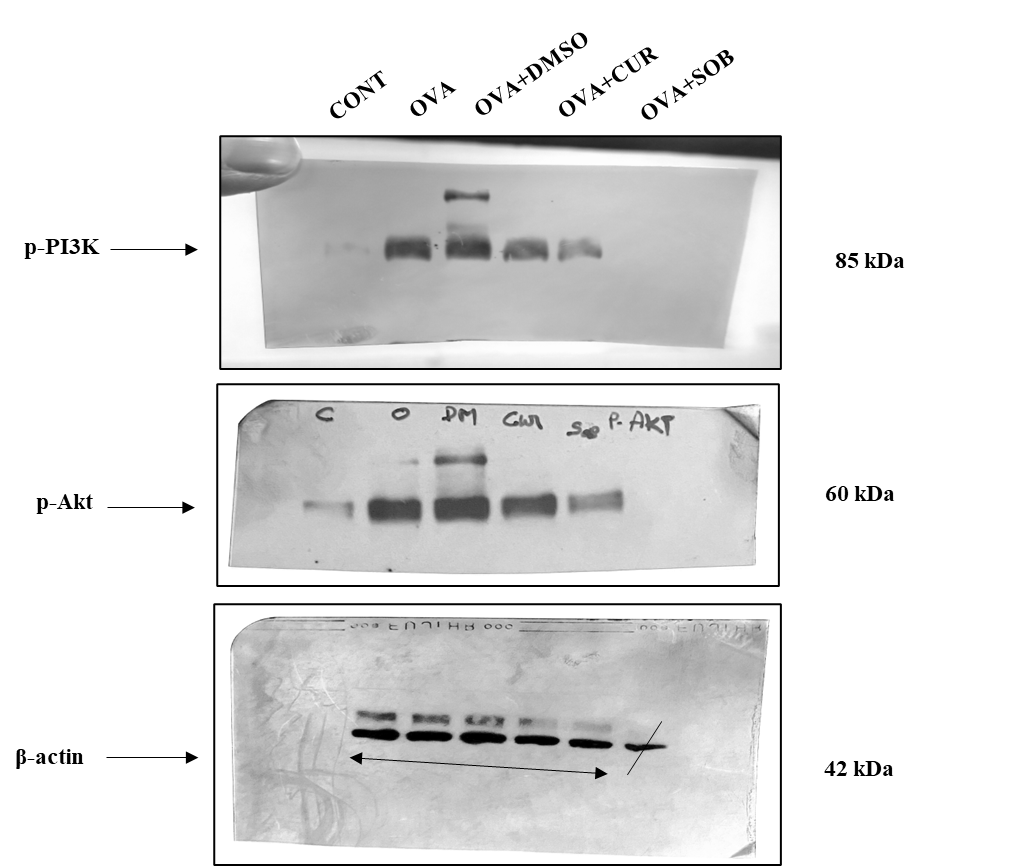


**Supplementary Figure S4**: Original whole western blot p-PI3K, p-Akt and β-actin. Membranes of p-PI3K and p-Akt were cut prior to incubation with primary antibodies and lower part was used for incubation with other antibodies. For the final figure, the bands were cropped and adjusted so that the bands appear better. The whole horizontal line of bands was adjusted. They were then placed in the Figure 9.


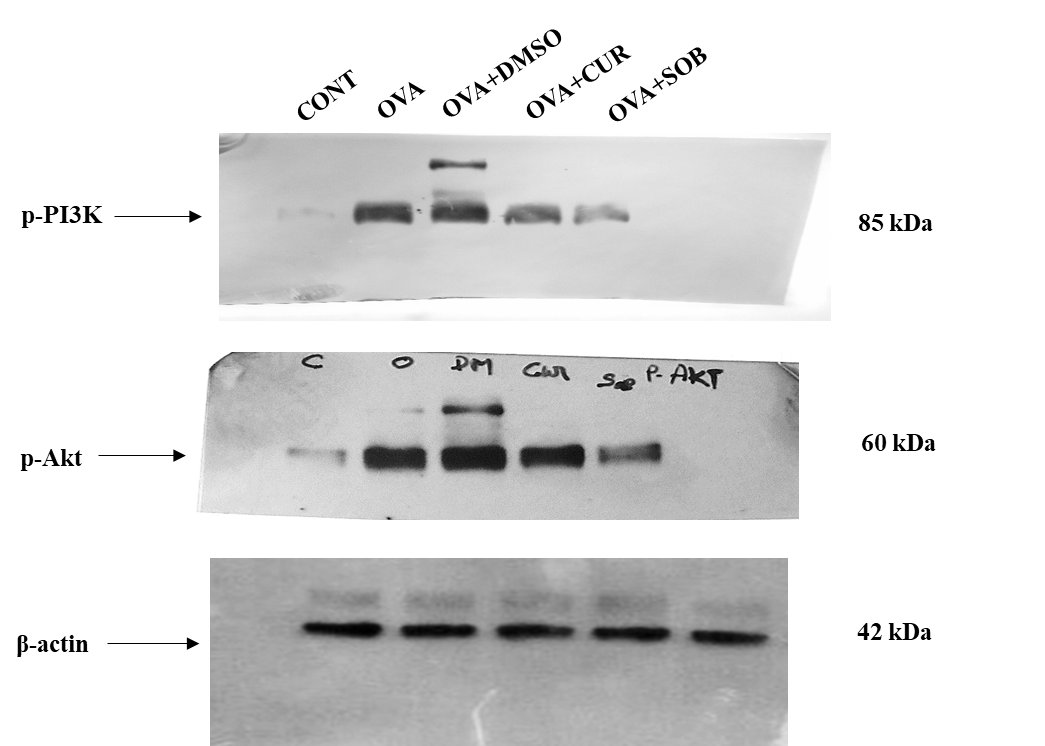


**Supplementary Figure S4:** Original whole western blot p-PI3K, p-Akt and β-actin. For the final figure, the bands were cropped and adjusted so that the bands were more apparent. The whole horizontal line of bands was adjusted at the same time. They were then placed in the Figure 9.
